# Validation of the Dutch version of the Swallowing Quality-of-Life Questionnaire (DSWAL-QoL) and the adjusted DSWAL-QoL (aDSWAL-QoL) using item analysis with the Rasch model: a pilot study

**DOI:** 10.1186/s12955-017-0639-3

**Published:** 2017-04-07

**Authors:** Ingeborg S. Simpelaere, Gwen Van Nuffelen, Marc De Bodt, Jan Vanderwegen, Tina Hansen

**Affiliations:** 1 University of Antwerp, Antwerp, Belgium; 2 VIVES University College, Bruges, Belgium; 3 Department of Speech-Language Pathology, AZ Delta Hospital, Menen, Belgium; 4 Department of Otolaryngology and Rehabilitation Centre for Communication Disorders, Antwerp University Hospital, Antwerp, Belgium; 5 Department of Translational Neurosciences, Faculty of Medicine and Health Sciences, University of Antwerp, Antwerp, Belgium; 6 Department of Speech, Language and Hearing Sciences, Faculty of Medicine and Health Sciences, Ghent University, Ghent, Belgium; 7 Department of Otolaryngology, Head and Neck Surgery, Saint-Pierre University Hospital, Brussels, Belgium; 8 Thomas More University College, Antwerp, Belgium; 9 Institute of Physiotherapy and Occupational Therapy, Metropolitan University College, Copenhagen, Denmark

**Keywords:** Deglutition disorders, Health-related quality of life, Oropharyngeal dysphagia, Outcome assessment, Psychometrics, Rasch model, SWAL-QoL

## Abstract

**Background:**

The Swallowing Quality-of-Life Questionnaire (SWAL-QoL) is considered the gold standard for assessing health-related QoL in oropharyngeal dysphagia. The Dutch translation (DSWAL-QoL) and its adjusted version (aDSWAL-QoL) have been validated using classical test theory (CTT). However, these scales have not been tested against the Rasch measurement model, which is required to establish the structural validity and objectivity of the total scale and subscale scores. Thus, the purpose of this study was to examine the psychometric properties of these scales using item analysis according to the Rasch model.

**Methods:**

Item analysis with the Rasch model was performed using RUMM2030 software with previously collected data from a validation study of 108 patients. The assessment included evaluations of overall model fit, reliability, unidimensionality, threshold ordering, individual item and person fits, differential item functioning (DIF), local item dependency (LID) and targeting.

**Results:**

The analysis could not establish the psychometric properties of either of the scales or their subscales because they did not fit the Rasch model, and multidimensionality, disordered thresholds, DIF, and/or LID were found. The reliability and power of fit were high for the total scales (PSI = 0.93) but low for most of the subscales (PSI < 0.70). The targeting of persons and items was suboptimal. The main source of misfit was disordered thresholds for both the total scales and subscales. Based on the results of the analysis, adjustments to improve the scales were implemented as follows: disordered thresholds were rescaled, misfit items were removed and items were split for DIF. However, the multidimensionality and LID could not be resolved. The reliability and power of fit remained low for most of the subscales.

**Conclusions:**

This study represents the first analyses of the DSWAL-QoL and aDSWAL-QoL with the Rasch model. Relying on the DSWAL-QoL and aDSWAL-QoL total and subscale scores to make conclusions regarding dysphagia-related HRQoL should be treated with caution before the structural validity and objectivity of both scales have been established. A larger and well-targeted sample is recommended to derive definitive conclusions about the items and scales. Solutions for the psychometric weaknesses suggested by the model and practical implications are discussed.

**Electronic supplementary material:**

The online version of this article (doi:10.1186/s12955-017-0639-3) contains supplementary material, which is available to authorized users.

## Background

Health-related quality of life (HRQoL) refers to a complex, multidimensional construct and is based on the individuals’ subjective perceptions of functioning and wellbeing among the physical, psychological and social domains of health [1–3]. The construct HRQoL is not directly measurable or is unobservable or latent [4] and should preferably be measured with patient-reported outcome (PRO) measures using multiple items, each assessing a different aspect of the underlying construct [5]. Many PROs have been developed to measure HRQoL in patients with oropharyngeal dysphagia [1]. These PROs use self-reported questionnaires, assess the presence and severity of dysphagia symptoms, and measure the influence of dysphagia on a person’s HRQoL [1, 6–8]. These PROs add useful information to the clinical swallowing examination and instrumental investigations [9] and can be used as outcome measures of therapeutic interventions [1, 6–8]. The applicability and appropriateness of a PRO in a specific population depend on the target population (i.e., persons with oropharyngeal dysphagia), its feasibility and the quality of its psychometric properties (i.e., reliability and validity) [4, 10].

The Swallowing Quality-of-Life questionnaire (SWAL-QoL) is a 44-item disease-specific scale that is distributed into 10 subscales and the Symptom scale [11]. The SWAL-QoL is considered the gold standard for assessing HRQoL in oropharyngeal dysphagia [12]. The psychometric properties of the SWAL-QoL as well as the Dutch translation of the SWAL-QoL (DSWAL-QoL) and its adjusted version (aDSWAL-QoL) have been demonstrated to be sufficient according to classical test theory (CTT) [7, 11, 13]. The CTT-psychometric assessment included an examination of internal consistency based on Cronbach’s alpha, test-retest reliability via the intraclass correlation coefficient (ICC), and/or construct validity based on principal component analysis (PCA) techniques [7, 11, 13]. Some drawbacks related to CTT methods are recognized [14, 15], such as test and sample dependence [14–16] and the assumption of equal weight for all of the items even if there is a difference in the level of difficulty [16]. The scale’s total sum score is based on ordinal values and the standard error of measurement is assumed to be constant [10, 15], in contrast to the Rasch methodology.

The Rasch model within modern item response theory (IRT) has been considered the gold standard against which scales summarizing item responses must be tested [17]. Item analysis using the Rasch model involves formal testing of a scale against a mathematic measurement model that specifies what should be expected in the item responses to provide interval-based measures instead of ordinal values [18, 19]. Interval measures are preferable to ordinal scales because they provide meaningful information about the relative differences and equivalences within the categories of the scale and enable the use of parametric statistics, which provide more powerful and precise results [14, 20]. If the observed data fit the model, the following can be concluded: interval data have been generated, the measurement scale demonstrates structural validity and objectivity, and the total score is statistically sufficient [17]. Structural validity is an aspect of construct validity and evaluates the extent to which the scores of a HRQoL-PRO are an adequate reflection of the dimensionality of the construct being measured [4]. Objectivity implies invariance, which indicates that the comparison between two persons should be independent of which particular items have been used and vice versa [21]; therefore, the instrument should work in the same manner across all persons and items. In contrast to CTT, Rasch measurements allow for the provision of scale-independent person estimates and sample-independent item estimates [5]. The total score of a scale is statistically sufficient if the assessment of the latent variable (i.e., HRQoL) is only a function of that total score and does not depend on the conditional distribution of the item responses underlying the total score [17]. Four assumptions should be satisfied for a measurement scale to meet the criteria of validity, objectivity and statistical sufficiency: 1) unidimensionality (all items in the scale measure the same single construct) [17, 21], 2) monotonicity (the scale items function hierarchically from easy to difficult, with increased item scores corresponding to increased levels of underlying ability) [17, 22], 3) local item independency (a person’s score on one item does not depend on their score on another item), and 4) no differential item functioning (DIF, i.e., a particular item’s score does not differ due to other factors, e.g., age, for persons with equal ability levels) [17, 21]. In addition to identifying measurement weaknesses, analysis with the Rasch model provides potential solutions for scale improvement. Such improvement has previously been demonstrated with the Taiwan Chinese version of the EORTC QLQ-PR25 questionnaire [23], St. George’s Respiratory Questionnaire (SGRQ) [16], and the Patient-Rated Elbow Evaluation (PREE) questionnaire [24].

The purpose of this study was to assess the structural validity and objectivity of both the DSWAL-QoL and aDSWAL-QoL scales and subscales and the statistical sufficiency of the total score and subscale scores using item analysis with the Rasch model.

## Methods

### Participants

A portion of the data was derived from a previous validation study of the aDSWAL-QoL, which has been extensively reported elsewhere [13]. Therefore, the design will be briefly described. A cross-sectional study using convenience sampling was conducted and included 108 persons, among whom 78 were involved in the previous study [13]. People were selected if they were (1) native Dutch speakers, (2) adults (age ≥18 years old) and (3) had oropharyngeal dysphagia of mechanical or neurological origin as assessed with the Mann Assessment of Swallowing Ability (MASA) [25] and/or the Fiberoptic Endoscopic Evaluation of Swallowing (FEES) [26]. Persons without oropharyngeal dysphagia but with a confirmed language and/or cognitive impairment as measured by the auditory and visual comprehension subtests of the Akense Afasie Test (AAT) [27] and the Mini Mental State Examination (MMSE) [28] were also included in this study. Persons were classified into three groups according to whether they suffered from dysphagia (Dys group), had dysphagia accompanied by a language impairment and/or cognitive disorder (DysLC group), or suffered from a language impairment and/or cognitive disorder without the presence of dysphagia (LC group). The proposed criteria for the MASA [25] (further specified in Table 1) and the standardized cut-off scores of 107 for the AAT [27] and 27 for the MMSE [29, 30] were used to compose the groups. The exclusion criteria were as follows: (1) severe problems understanding written and spoken Dutch resulting in the inability to complete the questionnaires; (2) severe attention and/or concentration problems that affected the person’s ability to maintain concentration during the assessment; (3) the presence of purely esophageal dysphagia; (4) anosognosia, i.e., being unaware of the existence of dysphagia despite clinical confirmation; and (5) severe visual and hearing impairments that prevented the investigators from successfully providing assistance when required. The people were recruited from different settings that included hospitals, rehabilitation centers, nursing homes and private speech-language pathologist (SLP) practices and were identified by SLPs, the appropriate staff in nursing homes and medical doctors based on the inclusion criteria. Verbal and written consent were obtained from the participants prior to the start of the study. Ethical approval for the consent procedure and the experimental protocol of the study was granted by the Committee for Medical Ethics of the Antwerp University Hospital and Antwerp University (B300201318058), and the study was conducted in full accordance with the Declaration of Helsinki.

**Table 1:** Demographic characteristics of the subjects (*N* = 108)

Characteristic	Dys (*N* = 35)		DysLC (*N* = 43)		LC (*N* = 30)	
Age (years)						
Mean (SD) (Min, Max)	62	(13.01) (35–89)	77	(11.04) (52–94)	81	(14.65) (20–95)
Gender, *N*, %						
Male	23	65.7	22	51.2	12	40.0
Female	12	34.3	21	48.8	18	60.0
Etiology, *N*, %						
Stroke	14	40.0	23	53.5	4	13.3
Head trauma					1	3.3
Head and neck cancer	17	48.6	2	4.7	0	0.0
Parkinson’s disease	1	2.9	6	14.0	4	13.3
Amyotrophic lateral sclerosis	1	2.9				
Multiple sclerosis	1	2.9				
Corticobasal degeneration	1	2.9				
Presbyphagia			8	18.6		
Dementia			3	7.0	18	60.0
Depression					3	10.0
Cerebral palsy			1	2.3		
Dysphagia (MASA)						
Mean (SD)	157.31	(16.68)	160.93	(10.27)	188.67	(5.41)
Dysphagia, *N*, %						
Severe (≤138)	7	20.0	1	2.3		
Moderate (≤139-167)	16	45.7	27	62.8		
Mild (≤168-177)	12	34.3	15	34.9		
Normal swallowing (≤178-200)	0	0	0	0	30	100.0
Aspiration, *N*, %						
Severe (≤140)	8	22.9	3	7.0		
Moderate (≤148)	3	8.6	3	7.0		
Mild (≤149-169)	15	42.9	31	72.1		
No aspiration (≤170-200)	9	25.7	6	14.0	30	100.0
MMSE						
Mean (SD) (Min, Max)	28.37	(1.11) (27–30)	20.35	(4.85) (5–28)	19.10	(4.77) (4–26)
AAT						
Visual Comprehension, Mean (SD)	56.09	(2.54)	39.84	(10.24)	38.77	(9.09)
Auditory Comprehension, Mean (SD)	55.43	(2.81)	36.37	(9.54)	34.90	(8.64)
Total score AAT, Mean (SD) (Min, Max)	111.54	(4.46) (107–120)	76.21	(17.69) (37–106)	73.67	(15.46) (31–95)
Highest completed education, *N*, %						
Primary school	14	40.0	30	69.8	18	60.0
High school	12	34.3	11	25.6	11	36.7
University college	7	20.0	2	4.7	1	3.3
University	2	5.7				
Place of living, *N*, %						
Home	27	77.1	6	14.0	3	10.0
Nursing home	4	11.4	30	69.8	23	76.7
Hospital	1	2.9	5	11.6	1	3.3
Rehabilitation center	3	8.6	1	2.3	3	10.0
Assisted living facility			1	2.3		

Abbreviations: *Dys* patients suffering from dysphagia, *DysLC* patients with dysphagia accompanied by language impairment and/or cognitive disorders, *LC* patients suffering from language impairment and/or cognitive disorders without the presence of dysphagia, *SD* standard deviation, *Min* Minimum, *Max* Maximum, *N* Number of persons, *%* percentage of people, *MASA* Mann Assessment of Swallowing Ability, *MMSE* Mini Mental State Examination, *AAT* Akense Afasie Test. Note that a portion of the data was published in a previous validation study of the aDSWAL-QoL [13]

### Measures

#### DSWAL-QoL and aDSWAL-QoL

The DSWAL-QoL is a condition-specific PRO scale that measures the effect of dysphagia on a person’s HRQoL [7]. The DSWAL-QoL has been validated for a Flemish population [7] and consists of 44 items that are grouped into the following 11 subscales: General burden, Eating desire, Eating duration, Symptoms, Food selection, Communication, Fear of eating, Mental health, Social functioning, Sleep and Fatigue. The DSWAL-QoL uses a 5-point Likert scale that ranges from 1 = ‘severely impaired quality of life’ to 5 = ‘no impairment’. Based on Likert’s method of summated ratings, the scores are transformed into subscale and scale scores that range from 0 = ‘strong effect of dysphagia on HRQoL’ to 100 = ‘no effect on HRQoL’ [31]. To increase the feasibility of the DSWAL-QoL for DysLC people, an adjusted version (aDSWAL-QoL) has been developed [13]. Both versions (DSWAL-QoL and aDSWAL-QoL) have been validated using CTT [7, 13]. The aDSWAL-QoL has similar content as the DSWAL-QoL (the abbreviated item contents of the DSWAL-QoL and aDSWAL-QoL are presented in Additional file 1) and also uses 5-point response categories. In contrast to the DSWAL-QoL, the number of different response formats in the aDSWAL-QoL is reduced to three (instead of six) and the subscales following the same response format are placed together. Additionally, the response categories in the aDSWAL-QoL are supported by visual line drawings, symbols and colors (Additional file 2).

#### Procedures

People completed both the DSWAL-QoL and aDSWAL-QoL in a random order to minimize recall effects. A minimum of 15 and a maximum of 30 min elapsed between the administration of the first and the second questionnaires. The people were encouraged to complete the scales as independently as possible, while assistance was provided when required (i.e., on request or when the patient failed to provide a response) [13].

#### The Rasch model

The Rasch model is based on a probabilistic Guttman pattern [10, 18]. This indicates that the probability of affirming a certain response to an item is a logistic function of the difference between the level of the measured construct as expressed by the person and as represented by the item and only a function of that difference [18, 21]. Person and item parameter estimates are placed on the same linear logit scale by transforming the original ordinal raw data into equal interval level measures (logits or log-odd units). The logit scale represents the latent trait [18] (i.e., dysphagia-related HRQoL) and both parameters are centered around a mean item location of zero [21]. Positive values for the person and item parameters indicate high ability levels (i.e., better HRQoL) and difficult items, and negative values indicate low ability levels (i.e., worse HRQoL) and easy items [32].

#### Data analysis

The DSWAL-QoL and aDSWAL-QoL include polytomous variables, and significant likelihood ratio tests (*p* < 0.001) indicated that the unrestricted parameterization of the model (partial credit) should be used rather than the rating scale model [33]. The item analysis with the Rasch model was performed using RUMM2030 [34], which integrates a pairwise conditional maximum likelihood algorithm in the estimation of the item and person parameters [22]. The following properties were examined: overall fit to the model, internal consistency reliability, unidimensionality, threshold ordering, individual item and person fits and differential item functioning (DIF), local item dependency (LID) and targeting. Item analysis with the Rasch model also yielded an iterative process in which strategies such as rescaling the response categories and item reductions were applied to improve the model fit and the construction of the scale.

##### Overall fit to the model

The overall fit to the model was assessed by evaluating three overall fit statistics, specifically two item-person interaction statistics and one item-trait interaction statistic [35]. The overall item and person fit were evaluated by inspecting the mean item and mean person standardized fit residuals (FRs) [35], which should be close to zero with a standard deviation (SD) of ‘<1.4’ [32]. The item-trait interaction that assesses whether the relative difficulties of the items remained constant across the different ability groups of patients [32, 35] was measured using a chi-square statistic (*χ* ^2^). Specifically, the *χ* ^2^ summarizes the differences between the observed and the expected values and was considered to be non-significant (*p* > 0.05) to fit the model expectations.

##### Reliability

The internal consistency reliability was assessed with the Person Separation Index (PSI), which is an estimate similar to Cronbach’s alpha (α) coefficient [35]. The PSI assesses how adequately the set of items can distinguish subjects on different levels of the scale [36], and a value ≥ 0.70 is required [18, 32]. The PSI is also an indicator of the power of the generated fit statistics [24].

##### Unidimensionality

The unidimensionality of the scale was measured by performing t-tests on the two most divergent subsets of items [32]. The items with the greatest positive and negative loadings on their first residual factor (resulting from PCA) were used to create the two subsets [32, 37]. The scale was considered unidimensional if < 5% of the person estimates exhibited a significant difference in the scores for the two subtests [32, 37] or if the lower bound of an exact binomial confidence interval is < 5% [19]. Performing the *t*-test requires at least 12 category thresholds in each of the two subsets [19].

##### Threshold ordering of the polytomous items

**Fig. 1:**
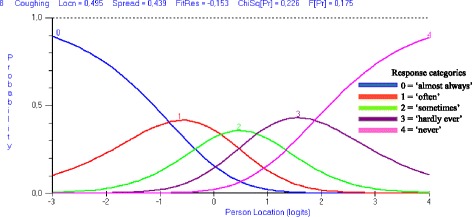
Category probability curve with ordered thresholds. Category probability curve displaying ordered thresholds for item 8 of the total DSWAL-QoL scale. This item has five response categories, resulting in 4 thresholds that increase in their location on the latent trait in a manner consistent with the increase in the underlying trait being measured. Note that the original response category structure of ‘1 to 5’ was transformed into ‘0 to 4’ by the Rumm software. Each response category (0, 1, 2, 3, 4) has a point (indicated by a peak in the curve) along the latent trait at the point of the most probable response

After the investigation of the overall fit statistics, the ordering of the response categories was examined using a threshold map and category probability curves [18, 21]. In the cases of both the DSWAL-QoL and aDSWAL-QoL items, there are 5 response categories, resulting in 4 thresholds (= transitional points) [14]. Figure 1 represents a category probability curve in which the thresholds of a certain item are well ordered and form distinctive regions. Monotonicity was expected, and in cases of disordered thresholds, the item was rescored by combining adjacent categories [18, 32].

##### Individual item and person fit

Individual item and person fit were assessed using the FR and χ^2^. A person and item FR ± 2.5 and a χ^2^ statistic above a Bonferroni-adjusted α-value of 0.05 [32, 38] indicated a fit to the model. Misfitting items or persons were removed to improve the overall fit of the model.

##### Differential item functioning

Items were also checked for DIF to ensure that the items of the scale were not biased by the person factors (i.e., language and cognitive impairments and dysphagia) and that the different class intervals followed the expected values of the characteristics of the items themselves. Thus, it was possible to investigate whether the different groups of the sample responded differently to an individual item despite the equal location on the latent trait [21]. The detection of DIF (i.e., uniform [18] and non-uniform DIF [18, 21, 37]) was made possible via the application of analysis of variance (ANOVA) to the fit residuals [21]. Uniform DIF was adjusted by splitting the item into group-specific items [21]. Items with non-uniform DIFs were considered to misfit the model and were removed [35].

##### Local item dependency

Local item dependency, which might be caused by response dependency (i.e., when a person’s response to an item depends on the response to another item) or by trait dependency (i.e., multidimensionality), was investigated using the residual correlation matrix [22, 39]. Local item dependence was considered to be present if the item residual correlations were > 0.3 above the average of all of the item residual correlations [22, 38]. By grouping the items into one “super-item,” called a testlet, the LID can be adjusted [37]. For all analyses, the Bonferroni correction was applied to adjust for multiple testing and was calculated based on the number of items [21].

##### Targeting of persons and items (person-item threshold distributions)

Targeting was examined after fitting the best solutions for the DSWAL-QoL and aDSWAL-QoL total scales and subscales. Targeting was analyzed by comparing the person and item threshold distributions. To be acceptable, the mean person locations were expected to approximate the mean item threshold location (i.e., 0.0 logits) and the item locations were expected to cover approximately the same range of the logit scale as the person locations [21, 35].

#### Sample size

A sample size of 108 persons was suggested to provide 95% confidence that the item calibration or the estimated item difficulty will be within ± 0.5 logits [40].

## Results

### Participants

In total, 108 persons were included. The Dys group consisted of 35 persons, 43 persons comprised the DysLC group, and 30 persons were in the LC group. The mean age of the total sample was 73.50 years (SD: 14.79). Table 1 presents the demographic characteristics of the persons. Comparison of the three groups revealed that head and neck cancer (48.6%) were most common in the Dys group, stroke (53.5%) was most common in the DysLC group, and dementia (60.0%) was most common in the LC group.

### Evaluation of the measurement properties of the DSWAL-QoL and aDSWAL-QoL

Tables 2 and 4 display the results for the overall fit statistics before and after the implementation of the solutions suggested by the Rasch model for the DSWAL-QoL and aDSWAL-QoL scales, respectively. Table 3 provides an overview of the item level fit statistics of both scales.

**Table 2:** Overall fit statistics for the DSWAL-QoL scale

Analysis		Item-person interaction		Item-trait interaction		Reliability	Unidimensionality	% ext
Scale	Initial scale (# items) Rescaled scale (suggestions by the Rasch model to improve model fit) (# items after adjustments)	Item FR Mean (SD)	Person FR Mean (SD)	*χ* ^2^ (df)	p	PSI +ext/÷ext	t-test %, (95% CI)	
Total scale	IS Total scale (44)	0.43 **(1.80)**	0.18 **(1.62)**	181.03 (44)	**<0.001**	0.93/0.93	***16.09 (10.9-19.2)***	0
	RS Total scale (rescore 41 items, delete 6 misfit items) (38)	0.03 (1.11)	−0.28 **(1.87)**	55.47 (38)	**0.032**	0.93/0.93	***15.24 (11.1-19.4)***	0.9
General burden	IS General burden (2)	0.33 (0.15)	−0.49 (1.0)	3.37 (2)	0.185	0.33/-0.61	NA	39.8
	RS General burden (rescore 2 items) (2)	0.70 (0.17)	−0.88 **(1.66)**	0.49 (2)	0.782	0.17/-2.64	NA	65.0
Eating duration	IS Eating duration (2)	0.39 (0.29)	−0.32 (1.05)	4.38 (2)	0.112	0.18/-1.32	NA	45.1
	RS Eating duration (rescore 2 items) (2)	0.96 (0.29)	**−1.59 (3.00)**	3.82 (2)	0.148	−0.10/-3.47	NA	52.9
Eating desire	IS Eating desire (3)	1.08 (0.72)	−0.10 (**1.46**)	8.74 (3)	**0.033**	0.13/-0.51	NA	30.8
	RS Eating desire (rescore 3 items) (3)	1.11 (0.56)	−0.73 **(2.72)**	8.85 (3)	**0.031**	0.28/-0.69	NA	30.8
Symptoms	IS Symptoms (14)	0.04 (0.94)	−0.17 (1.16)	13.66 (14)	0.475	0.83/0.84	***9.3 (4.9-13.4)***	6.6
	RS Symptoms (rescore 11 items) (14)	0.01 (1.05)	−0.21 (1.07)	18.91 (14)	0.168	0.85/0.84	***8.2 (3.8-12.6)***	6.6
Food selection	IS Food selection (2)	0.21 (0.04)	−0.39 (0.66)	2.96 (2)	0.227	0.46/0.20	NA	40.6
	RS Food selection (rescore 2 items) (2)	0.47 (0.21)	−0.70 (1.28)	0.77 (2)	0.682	0.54/0.25	NA	40.6
Communication	IS Communication (2)	0.41 (0.06)	−0.70 (1.06)	0.19 (2)	0.908	0.77/0.48	NA	35.6
Fear of eating	IS Fear of eating (4)	0.32 (0.96)	−0.30 (1.14)	16.90 (4)	**0.002**	0.55/0.41	NA	25.5
	RS Fear of eating (rescore 4 items, DIF-split 1 item) (5)	0.17 (0.92)	−0.45 (1.22)	8.05 (5)	0.153	0.54/0.38	NA	25.5
Mental health	IS Mental health (5)	0.20 (0.76)	−0.41 (1.35)	4.45 (5)	0.487	0.75/0.75	NA	30.5
	RS Mental health (rescore 1 item) (5)	0.26 (0.79)	−0.37 (1.23)	2.33 (5)	0.801	0.74/0.74	NA	30.5
Social functioning	IS Social functioning (5)	0.17 (0.64)	−0.41 (1.17)	5.09 (5)	0.405	0.72/0.59	NA	31.1
	RS Social functioning (rescore 5 items) (5)	0.40 (0.92)	−0.39 **(1.44)**	8.19 (5)	0.146	0.72/0.44	NA	36.8
Fatigue	IS Fatigue (3)	0.50 (0.11)	−0.52 (1.10)	2.30 (3)	0.513	0.69/0.56	NA	20.8
	RS Fatigue (rescore 3 items) (3)	0.49 (0.23)	−0.26 (0.88)	1.27 (3)	0.737	0.61/0.38	NA	30.2
Sleep	IS Sleep (2)	0.86 (0.06)	−0.54 (1.22)	2.13 (2)	0.345	0.27/-0.88	NA	43.4
	RS Sleep (rescore 2 items) (2)	0.47 (0.05)	**−1.63 (1.90)**	0.63 (2)	0.729	0.17/-2.40	NA	43.4
Satisfactory fit		0.00 (<1.40)	0.00 (<1.40)		>0.05	≥0.70	<5% or LCI ≤5%	

Abbreviations and symbols: *DSWAL-QoL* Dutch version of the Swallowing Quality-of-Life, *IS* initial scale, *RS* rescaled scale based on the suggestions from the Rasch methodology, *DIF* differential item functioning, *FR* fit residual, *SD* standard deviation, *PSI* Person separation index with extremes (+) and without extremes (÷), *χ* ^*2*^ *(df)* chi-square (degrees of freedom), *LCI* lower confidence interval, *% ext* percentage of extreme scores, *NA* not applicable (T-tests were not performed when there were too few thresholds in each subset or when the items were split for DIF when using the RUMM software). Bold indicates misfit to the Rasch model. Bold italic indicates multidimensionality

**Table 3:** Item analysis: Overview of the item-level fit statistics of the DSWAL-QoL and aDSWAL-QoL

Scale (# items)	Disordered thresholds		Misfitting items		DIF		LID	
	DSWAL-QoL Item No	aDSWAL-QoL Item No	DSWAL-QoL Item No	aDSWAL-QoL Item No	DSWAL-QoL Item No	aDSWAL-QoL Item No	DSWAL-QoL Item No	aDSWAL-QoL Item No
Total scale (44)	1–7,9,11–15,17, 19,22–44	1–7,10,11,13,14, 17–41,43	5: FR = 6.1^a^ 6: FR = 6.2^a^ 7: FR = 4.1^a^ 32: FR = −2.3^a^ 34: FR = −2.0^a^ 40: FR = 3.4^a^ 41: FR = 2.6	2: FR = −2.8^a^ 5: FR = 2.7^a^ 6: FR = 5.9^a^ 25: FR = 3.2 29: FR = 5.4^a^ 32: FR = −2.7^a^ 40: FR = 3.5^a^ 41: FR = 3.2	5^b^	1,2,6,9,32,43^b^	clusters within and across subscales	
General burden (2)	1,2							
Eating duration (2)	3,4	3				3^b^		
Eating desire (3)	5,6,7	5,6,7						
Symptoms (14)	9,11–19,20	9,11–15,17,19,20					8/9	9/10, 19/20
Food selection (2)	22,23	22,23						
Communication (2)		24						
Fear of eating (4)	26,27,28,29	26,27,28,29		29: FR = 3.2^a^	26^b^	26^b^		26/28
Mental health (5)	30	30,33,34				31^c^		
Social functioning (5)	35,36,37,38,39	35,36,37,38,39						
Sleep (2)	40,41	40						
Fatigue (3)	42,43,44							

Abbreviations: *DSWAL-QoL* Dutch version of the Swallowing Quality-of-Life, *aDSWAL-QoL* adjusted DSWAL-QoL, *No* number, *DIF* differential item functioning, *LID* local item dependence, *FR* fit residuals

^a^Indicates significant chi-square at *p* < 0.001

^b^Uniform DIF

^c^Non-uniform DIF

#### Evaluation of the measurement properties of the DSWAL-QoL

**Fig. 2:**
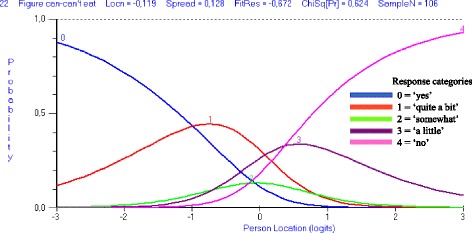
Category probability curve with disordered thresholds. Category probability curve graphically highlighting the disordered thresholds for item 22 of the total DSWAL-QoL scale. The point at which the lines for the adjacent response categories intersect in item 22 indicates that the transition between categories 2 and 3 is lower on the trait than the transition between categories 0 and 1. Response categories 2 and 3 never have a point on the continuum at which the most probable response is located

**Fig. 3:**
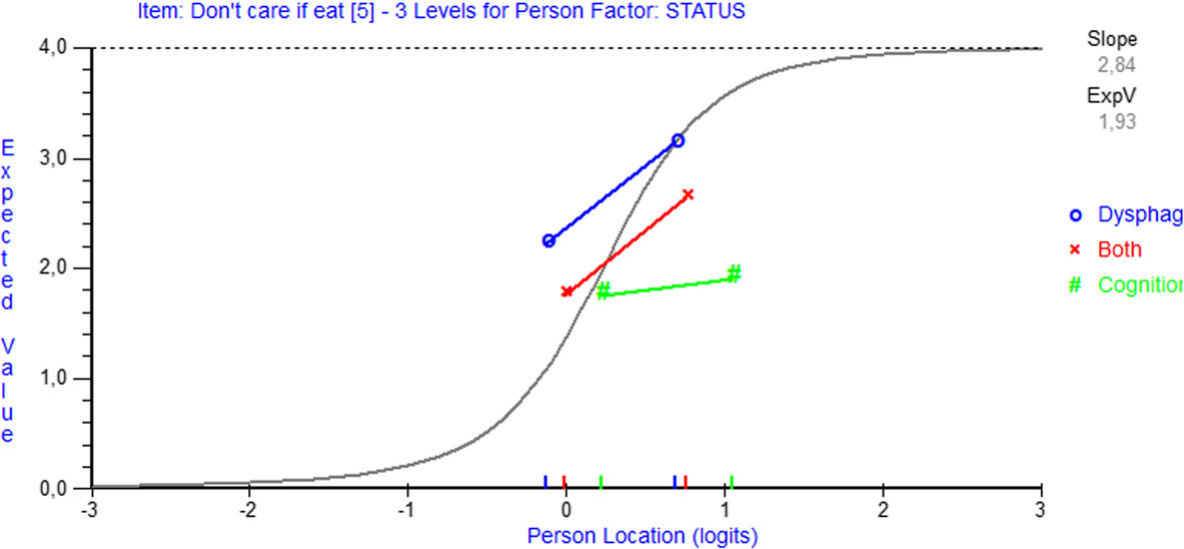
Item characteristic curve displaying a uniform DIF. Item characteristic curve displaying a uniform DIF for item 5. Despite the equal ability level, the three groups responded differently. The cognitive group obtained a prominently lower score than those of the two other groups

The analysis revealed that the reliability was good, with a PSI of 0.93 and an excellent power of fit without extreme scores. However, the total DSWAL-QoL scale was found to misfit to the Rasch model as indicated by the item FR SD and person FR SD > 1.4, and by the presence of a significant item-trait interaction (Table 2). Multidimensionality was present as confirmed by the 16.09% statistically significant different person estimates based on the two subsets of items. Disordered thresholds were found in 38 items, which indicated that the categorization of these items did not work as intended (Table 3). For example, the category probability curve for item 22 revealed that the estimates of the thresholds defining categories 2 and 3 did not form distinctive regions on the latent trait; therefore, these scores (i.e., ‘somewhat’ and ‘a little’) were at no time the most probable responses (Fig. 2). Seven items did not fit (items 5, 6, 7, 32, 34, 40 and 41; Table 3), and the individual person fit revealed that 11 persons fell outside the FR range of ± 2.5. As illustrated in Fig. 3, item 5 exhibited a uniform DIF by group for all three groups, and the cognitive group obtained a prominently lower score compared with the those of persons in the other two groups given an equal ability level. Residual correlations > 0.3 were found for clusters of items within and across subscales; thus, LID was present. To achieve a satisfactory overall model fit, it was necessary to rescore 38 items. For example, scores 2 and 3 for item 22 were collapsed into the score ‘1’; therefore, the Rasch-suggested scoring solution revealed a three-point response category, i.e., ‘0, 1 and 2,’ for this item (Additional file 1). During this process of rescoring items, three additional items exhibited disordered thresholds and were also rescored. It was also necessary to delete six items (items 5, 6, 7, 34, 40 and 41; Table 2). The overall model fit improved for the items and no further DIF was present. However, the overall fit worsened for the person FR SD, the item-trait interaction remained significant, and 14 persons did not fit. Due to the limited sample size, the misfit persons were not removed. Multidimensionality remained despite the adjustments, and LID could not be resolved due to its unclear pattern.

For the Symptoms and Mental health subscales, the reliabilities were acceptable (PSI ≥ 0.75; Table 2). For the other subscales, the reliabilities were below the recommended level and the power of fit was low. A large number of extreme scores were present for all subscales with the exception of the Symptoms scale. There was no pattern of the extremes across the three person groups. All of the DSWAL-QoL subscales, with the exception of the Eating desire and Fear of eating subscales, exhibited satisfactory overall fit statistics. For the Eating desire subscale, the person FR mean (SD) of −0.10 (1.46) indicated some misfit of the persons. Inspection of the individual person fits revealed five misfit persons. The Symptom subscale exhibited a lack of unidimensionality, whereas the other subscales could not be subjected to t-tests due to insufficient numbers of items (i.e., thresholds). None of the items exhibited misfit, but disordered thresholds were found for a majority of the items within all subscales with the exception of the Communication subscale (Table 3). A uniform DIF was identified for item 26 from the Fear of eating subscale and was biased toward the cognitive group, which obtained higher scores. Local item dependency was demonstrated between items 8 and 9 from the Symptoms subscale. Adjustments of the subscales were performed, and after the items with disordered thresholds were rescored and item 26, which exhibited DIF, was split, all of the items exhibited ordered thresholds, the LID disappeared and the item-trait interaction improved for the Fear of eating subscale (Table 2). However, the overall person FR mean values and/or SDs significantly increased for some of the subscales (i.e., General burden, Eating duration, Eating desire, Social functioning and Sleep), and the item-trait interaction remained significant for the Eating desire subscale. For the General burden, Eating duration, Eating desire, Social functioning and Sleep subscales, the numbers of misfit persons were *N* = 4, *N* = 14, *N* = 11, *N* = 7, and *N* = 33, respectively. Unidimensionality could not be established for the Symptom subscale, and improvement for reliability also could not be identified for the subscales. After the solutions, the numbers of extreme scores increased for the General burden, Eating duration, Social functioning and Fatigue subscales (Table 2). The misfit persons were not removed because of the limited sample size.

#### Evaluation of the measurement properties for the aDSWAL-QoL

The analysis of the total aDSWAL-QoL scale revealed that the reliability was good (PSI = 0.93), the power of fit was excellent, and there were no extreme scores. Nonetheless, the total aDSWAL-QoL scale significantly deviated from the Rasch model (Table 4). Approximately 18% of the person estimates on the two most divergent subsets of items were significantly different, which indicated multidimensionality. Individual item analysis revealed 37 items with disordered thresholds (Table 3). Eight items (items 2, 5, 6, 25, 29, 32, 40 and 41) exhibited individual item misfit, and six items (1, 2, 6, 9, 32 and 43) exhibited uniform DIFs (Table 3). Most of the items with DIF were biased toward the LC group, which obtained higher scores, with the exception of item 43. Individual person misfits were found for 16 persons. Local item dependency was present between several item pairs within and across the subscales. After the rescoring of 37 items and six more items that exhibited disordered thresholds during the iterative process, the individual item fits improved for items 2 and 25 and the DIFs disappeared for items 6, 9 and 43. The other misfit items and the items that exhibited DIF remained. The iterative process revealed that to improve the overall fit, it was necessary to remove items 5, 6, 7, 29, 32, 40 and 41 and to split five items that displayed uniform DIFs (items 1, 2, 27, 43 and 44; Table 4). The person FR still indicated some misfit among the persons (SD > 1.4). The misfit persons (*N* = 19) were not removed because of the relatively limited sample size. Assessing the unidimensionality of the scale was no longer possible because of the item split. Since the LID showed an unclear pattern, it was not possible to create testlets.

**Table 4:** Overall fit statistics for the aDSWAL-QoL scale

Analysis		Item-person interaction		Item-trait interaction		Reliability	Unidimensionality	% ext
Scale	Initial scale (#items) Rescaled scale (suggestions by the Rasch model to improve model fit) (# items after adjustments)	Item FR Mean (SD)	Person FR Mean (SD)	χ^2^ (df)	p	PSI +ext/÷ ext	*t*-test %, (95%CI)	
Total scale	IS Total scale (44)	0.19 **(1.95)**	−0.01 **(1.64)**	302.65 (44)	**<0.001**	0.93/0.93	***17.59 (13.5-21.7)***	0
	RS Total scale (rescore 43 items, delete 7 misfit items, DIF-split 5 items) (42)	−0.19 (0.95)	−0.46 **(1.86)**	50.96 (42)	0.137	0.92/0.92	NA	0
General burden	IS General burden (2)	0.17 (0.10)	−0.57 (0.86)	1.58 (2)	0.453	0.69/0.51	NA	38.9
Eating duration	IS Eating duration (2)	−0.12 (0.32)	−0.29 (0.62)	3.95 (2)	0.139	0.70/0.42	NA	24.5
	RS Eating duration (rescore 1 item + DIF-split 1 item) (3)	0.06 (0.16)	−0.29 (0.60)	2.70 (3)	0.440	0.68/0.37	NA	24.5
Eating desire	IS Eating desire (3)	0.32 (0.51)	−0.24 (0.80)	6.17 (3)	0.104	0.61/0.40	NA	2.8
	RS Eating desire (rescore 3 items) (3)	0.37 (0.35)	−0.22 (0.73)	10.75 (3)	**0.013**	0.57/0.32	NA	10.2
Symptoms	IS Symptoms (14)	−0.05 (0.88)	−0.29 (1.25)	10.23 (14)	0.741	0.87/0.86	***10.8 (6.6-15.0)***	2.8
	RS Symptoms (rescore 9 items) (14)	−0.08 (0.64)	−0.29 (1.17)	12.02 (14)	0.605	0.86/0.85	***11.8 (7.5-16.0)***	2.8
Food selection	IS Food selection (2)	0.74 (0.77)	−0.10 (0.67)	5.21 (2)	0.074	−0.44/-1.31	NA	29.9
	RS Food selection (rescore 2 items) (2)	0.82 (0.78)	−0.10 (0.55)	1.11 (2)	0.574	−0.39/-1.33	NA	29.9
Communication	IS Communication (2)	0.25 (0.24)	−1.09 **(1.57)**	2.44 (2)	0.295	0.46/0.18	NA	18.1
	RS Communication (rescore 1 item) (2)	0.39 **(1.47)**	−1.14 **(1.89)**	1.12 (2)	0.570	0.43/-0.03	NA	27.6
Fear of eating	IS Fear of eating (4)	0.82 **(1.65)**	−0.36 (1.37)	57.75 (4)	**<0.001**	0.26/0.23	NA	3.8
	RS Fear of eating (rescore 2 items + delete 1 misfit item) (3)	0.66 (0.58)	−0.19 (1.06)	7.01 (3)	0.072	0.15/-0.31	NA	38.6
Mental health	IS Mental health (5)	−0.02 (0.83)	−0.48 (1.20)	4.46 (5)	0.486	0.71/0.72	NA	22.6
	RS Mental health (rescore 4 items) (5)	0.08 (0.91)	−0.41 (1.02)	6.66 (5)	0.247	0.73/0.72	NA	24.5
Social functioning	IS Social functioning (5)	0.08 (0.70)	−0.24 (0.96)	11.93 (5)	**0.036**	0.50/0.39	NA	31.8
	RS Social functioning (rescore 5 items) (5)	0.35 (0.81)	−0.49 **(1.49)**	3.801 (5)	0.578	0.62/0.46	NA	31.8
Fatigue	IS Fatigue (3)	0.12 (0.66)	−0.49 (1.03)	1.64 (3)	0.651	0.70/0.60	NA	14.3
Sleep	IS Sleep (2)	0.37 (0.10)	−0.38 (0.82)	3.15 (2)	0.207	0.43/0.03	NA	29.5
	RS Sleep (rescore 1 item) (2)	0.13 (1.40)	−0.29 (0.72)	2.50 (2)	0.287	0.39/-0.06	NA	35.2
Satisfactory fit		0.00 (<1.40)	0.00 (<1.40)		>0.05	≥0.70	<5% or LCI ≤5%	

Abbreviations and symbols: *aDSWAL-QoL* adjusted DSWAL-QoL, *IS* initial scale, *RS* rescaled scale based on the suggestions from the Rasch methodology, *DIF* differential item functioning, *FR* fit residual, *SD* standard deviation, *PSI* Person separation index reported with extremes (+) and without extremes (÷), *χ* ^*2*^ *(df)* chi-square (degrees of freedom), *LCI* lower confidence interval, *% ext* percentage of extreme scores, *NA* not applicable (T-tests were not performed when there were too few thresholds in each subset or when the items were split for DIF when using the RUMM software). Bold indicates misfit to the Rasch model. Bold italic indicates multidimensionality

For the Symptoms and Mental health subscales, the reliabilities were acceptable (PSI ≥ 0.72; Table 4). For the other subscales, the reliabilities were below the recommended level and the power of fit was low. Extreme persons were identified in all subscales, although the magnitudes were lowest for the Eating desire, Symptoms and Fear of eating subscales. Again, there was no pattern of extremes across the three population groups. The overall fits to the model were demonstrated for all of the aDSWAL-QoL subscales with the exception of the Communication, Fear of eating and Social functioning subscales (Table 4). The overall person FR indicated a misfit for the Communication subscale (SD = 1.57), and further analysis revealed 22 misfit persons. Multidimensionality was present for the Symptom subscale; however, the other subscales could not be subjected to the test for multidimensionality because there were too few items. Disordered thresholds were found for 29 items across all subscales with the exception of the items in the General burden and Fatigue subscales (Table 3). At the individual item level, item 29 of the Fear of eating subscale did not fit the model. Differential item functioning was found in 3 items (items 3, 26, and 31) and LID was found between two item pairs from the Symptoms subscale (9–10, 19–20) and between item pairs 26–28 from the Fear of eating subscale. After rescoring all of the items with disordered thresholds (the DIFs for items 26 and 31 disappeared after rescaling), removing the misfit item 29 and splitting item 3 for DIF, ordered thresholds were found for all items and the item-trait interactions improved for the Fear of eating and the Social functioning subscales (Table 4). The item FR SD increased for the Communication subscale and the item-trait interaction became significant for the Eating desire subscale (*p* < 0.05). The person FR SDs increased for the Communication and Social functioning subscales. The numbers of misfit persons were *N* = 21 and *N* = 9 for the Communication and Social functioning subscales, respectively, and misfit persons were not removed because of the relatively limited sample size. The number of extreme scores remained unchanged or increased. Improvement for reliability could not be identified for the subscales. The LID disappeared between items 26 and 28 from the Fear of eating subscale. For the Symptom subscale, the lack of unidimensionality remained and the LID persisted between items 9 and 10, disappeared between items 19 and 20 and appeared between items 14 and 18. Adjusting the LID (i.e., creating two testlets: item pair 9–10 and item pair 14–18) did not improve the overall fit statistics for the Symptom subscale (item FR (SD) = −0.10 (0.64); person FR (SD) = −0.32 (1.16); item-trait interaction: χ^2^ (df) = 12.16 (12); *p* = 0.433); however, the reliability increased (PSI = 0.98).

#### Targeting of persons and items

After the adjustments, targeting was suboptimal for both the DSWAL-QoL and aDSWAL-QoL total scales and subscales (Table 5). For both the total scales and subscales, the item locations did not cover the same ranges of the logit scale as the person locations. At the positive and negative ends of the trait, no item thresholds were found at the person locations, which indicated that these persons exhibited higher or lower ability levels that could not be measured by the items of the scale. For the total aDSWAL-QoL scale and the aDSWAL-QoL Symptoms subscale, at the negative end of the trait, no persons were located at the item thresholds, which indicated that the average item difficulties of some of the items were too low.

**Table 5:** Targeting of the DSWAL-QoL and aDSWAL-QoL total scales and subscales after fitting solutions

	DSWAL-QoL				aDSWAL-QoL			
	Item location		Person location		Item location		Person location	
	Mean (SD)	Range	Mean (SD)	Range	Mean (SD)	Range	Mean (SD)	Range
Total scale	0.0 (0.76)	−2.06; 1.20	0.89 (1.30)	−3.43; 5.36	0.0 (0.80)	−1.98; 2.05	1.05 (1.35)	−1.68; 5.98
General burden	0.0 (0.28)	−0.19; 0.19	−0.43 (1.52)	−1.92; 2.00	0.0 (0.28)	−0.15; 0.15	−0.43 (1.52)	−3.30; 3.01
Eating duration	0.0 (0.01)	−0.01; 0.01	−0.30 (1.20)	−1.65; 1.65	0.0 (0.72)	−0.61; 0.79	−0.80 (2.16)	−4.97; 2.59
Eating desire	0.0 (1.15)	−0.13; 0.16	0.15 (1.16)	−1.89; 1.89	0.0 (1.25)	−1.09; 1.37	0.53 (1.54)	−3.52: 3.78
Symptoms	0.0 (1.63)	−1.19; 0.79	1.32 (1.48)	−1.98; 4.60	0.0 (0.70)	−1.52; 0.96	1.07 (1.25)	−1.15; 4.63
Food selection	0.0 (1.22)	−0.16; 0.16	1.35 (2.00)	−2.92; 3.48	0.0 (0.87)	−0.61; 0.61	1.14 (1.67)	−2.85; 2.58
Communication	0.0 (1.34)	−0.24; 2.40	0.26 (2.56)	−4.04; 3.81	0.0 (0.04)	−0.03; 0.03	0.36 (1.41)	−2.37; 2.32
Fear of eating	0.0 (0.80)	−1.30; 0.27	1.42 (1.72)	−3.12; 3.92	0.0 (0.48)	−0.56; 0.28	0.98 (1.20)	−2.51; 2.19
Mental health	0.0 (0.28)	−0.24; 0.44	0.97 (1.63)	−3.00; 2.94	0.0 (0.52)	−0.72; 0.47	1.58 (1.77)	−3.41; 3.52
Social functioning	0.0 (0.47)	−0.80; 0.42	0.08 (1.89)	−2.89; 2.77	0.0 (0.53)	−0.85; 0.58	1.08 (1.61)	−3.09; 3.01
Fatigue	0.0 (0.37)	−0.41; 0.30	0.54 (1.78)	−3.00; 2.78	0.0 (0.32)	−0.26; 0.35	0.59 (1.45)	−3.14; 3.04
Sleep	0.0 (0.08)	−0.06; 0.06	0.33 (1.46)	−2.17; 2.17	0.0 (0.62)	−0.44; 0.44	0.48 (1.38)	−1.80; 2.27

Abbreviations: *DSWAL-QoL* Dutch version of the Swallowing Quality-of-Life, *aDSWAL-QoL* adjusted DSWAL-QoL, *SD* standard deviation

## Discussion

The analysis did not support the structural validity or objectivity of either the DSWAL-QoL or the aDSWAL-QoL total scales and subscales or the statistical sufficiency of the total scores and subscale scores. Misfit to the Rasch model, multidimensionality and/or the presence of DIF were found. Comparing the subscales of both versions, the Eating desire subscale of the aDSWAL-QoL exhibited an overall fit to the model in contrast to its corresponding subscale in the DSWAL-QoL, while the Communication and Social functioning subscales in the DSWAL-QoL did fit the model. For all other subscales, the results for the overall fits were similar. A large number of extreme scores were present in both versions of the scale, and this phenomenon was even greater for the DSWAL-QoL subscales. These extreme scores influenced the reliability and the power of fit. The presence of low levels of PSI and the high percentage of extreme scores reflected suboptimal targeting for the subscales of both versions. The suboptimal targeting for the total scales and subscales resulted in decreased estimation precisions of the item and person parameters [21]. The misfit items were most present when all of the items were treated as one total scale and were quite similar in both versions. Local item dependence was present between items within and across the subscales for both the DSWAL-QoL and aDSWAL-QoL scales.

The main sources of misfit for both scale versions were disordered thresholds for the items in the total scale and the individual subscales with the exception of the items of the Communication subscale in the DSWAL-QoL and the General burden and Fatigue subscales in the aDSWAL-QoL. We expected fewer disordered thresholds in the aDSWAL-QoL because the aDSWAL-QoL has been proven to be more feasible for use in groups with additional language and/or cognitive impairments [13]. Note that the 5-point response category in the aDSWAL-QoL contains similar content as the DSWAL-QoL. Some patients might have interpreted the graphic support (i.e., the symbols that were intended to enhance the comprehension of the response categories) in a different manner than what was intended. Nonetheless, it was obvious that the original scoring structures for most of the items of both the total scales and subscales did not work as intended (Additional file 1). The latter may be because the people were not able to discriminate between the response categories. Either the different categories were not well defined or the difference in meaning was too subtle (i.e., what is the difference between ‘somewhat’ and ‘a little’?). Additionally, the incorrect assumption that the Likert scale is an interval scale is common, although the categories of a 5-point Likert format represent a qualitative variable that is actually only sequential and ordinal [41]. To obtain linear, equal-interval level results, testing of the ordering of the response categories against the Rasch measurement model and subsequent rescaling of the items with disordered thresholds is required.

The presence of DIF by group was found in both versions of the scale but was most prominent for the aDSWAL-QoL. Most of the items that displayed DIF were biased toward the cognitive group, which tended to obtain higher scores for these items. This finding indicates that this group overestimated their HRQoL. The latter was expected because this group did not suffer from oropharyngeal dysphagia. The DSWAL-QoL and aDSWAL-QoL are disease-specific scales developed for people with oropharyngeal dysphagia. The LC group did not meet this condition; thus, the appropriateness of including this group in the study could be questioned. The objectivity of the scale can only be established by the Rasch methodology if one important requirement is satisfied, i.e., if the items and the sample are within the specific frame of reference for which the scale was developed [17]. Nonetheless, including this patient group was important because it enabled the evaluation of whether the scale and subscales were influenced by DIF. It was also expected that this LC group would exhibit extreme scores (i.e., all of the maximum scores for HRQoL) because of the absence of oropharyngeal dysphagia. However, there was no pattern in the extreme scores across the three groups. This issue leads to the question of the extent to which the scales are completed in a ‘reliable’ manner (i.e., whether they truly capture the patient’s perspective). Compared to the other two groups, more people in the cognitive group had an underlying etiology of dementia. The language and cognitive impairments were also greater in the LC group; thus, this group likely had more problems understanding the questions of both the DSWAL-QoL and aDSWAL-QoL. Next to impaired language functions, dementia encompasses a large spectrum of behavioral and other cognitive impairments, such as changes in personality and behavior, impaired reasoning and handling of complex tasks, poor decision-making ability and poor judgment [42]. The finding that this LC group did not demonstrate extreme scores as expected indicates that caution should be exercised in the use of these scales with dysphagic people with dementia because the dementia-related factors might influence the responses.

The use of the 5-point response category for the Social functioning subscale of the aDSWAL-QoL should be questioned because the middle response category of this subscale included ‘I don’t know.’ The literature indicates that respondents do not interpret this type of middle response category as expected from the integer scoring (i.e., monotonically). Consequently, disordered thresholds can occur because these categories differ from other response options in their probabilities of being selected. With respect to the integer scoring [43], it would be appropriate to reformulate this response category.

After the adjustments, a potential scoring structure was suggested for all items of both the DSWAL-QoL and aDSWAL-QOL total scales and subscales (Additional file 1). The scoring structure was often different for some of the items when they were treated as one scale instead of being part of the subscales. In most of the items, the 5-point response category was rescaled to a 3- or 4-point response format. A disadvantage of using different response formats is that it might lead to confusion and cause erroneous responses [44]. The analysis suggested that, rather than including 44 items, the total DSWAL-QoL scale should be rescaled to a 38-item scale and the total aDSWAL-QoL should be rescaled to a 42-item scale in which five items are group specific. The proposed numbers of items for the subscales of both versions are displayed in Additional file 1 and in Tables 2 and 4. Note that a large-scale empirical study is needed to confirm the scoring structures of both the scales and subscales.

The overall fit improved for the total aDSWAL-QoL scale but not for the total DSWAL-QoL after the adjustments, and the person FR SDs remained high for both total scales. Misfits were also demonstrated for the Eating desire subscale of both scales after adjusting the items. The issue of fit is a relative matter in the Rasch methodology because it depends on the sample size [21]. The reliabilities were high for both total scales but low for most of the subscales of both questionnaires. When comparing studies that performed cross-cultural adaptations of the SWAL-QoL [7, 8, 13, 45], we observed differences in reliability. These studies used Cronbach’s α to evaluate the internal consistency. However, relying on Cronbach’s α is only justified if the data are normally distributed. Multiple ceiling or floor effects were observed in those studies [7, 8, 13, 45], raising questions about the accuracy of the internal consistencies of these scales. It is not possible to compare our study results (based on the PSI) with studies that have used Cronbach’s α because α includes extreme scores, whereas the estimate of the PSI requires extrapolated values for extreme scores [46]. Specifically, the calculation of Cronbach’s α assumes equal standard errors (SEs) in all of the scores. This assumption contrasts with the calculation of the PSI in which the SE increases as the scores become more extreme [46]. After adjusting for LID in the Symptom subscale of the aDSWAL-QoL, the reliability increased, which indicated multidimensionality [39]. Whether LID is response or trait dependent may be difficult to distinguish in polytomous analysis [39]. For both the DSWAL-QoL and aDSWAL-QoL total scales, LID was present between and across subscales. Thus, due to an unclear pattern of the LID, it was not possible to create testlets that would resolve LID. Since LID might be caused by multidimensionality, it could be suggested to reconsider the dimensional structure of both scales using factor analytic approaches. Although Vanderwegen et al. [7] performed traditional (linear) PCA on the DSWAL-QoL, PCA only identifies the variables that show the strongest linear relationship with each other and tries to explain for as much of the total variance in the data [20]. Therefore, factor analysis for ordinal data [47] is required as it identifies the (number of) latent constructs and the possible underlying factor structure of a set of variables [20]. Furthermore, multidimensionality could not be resolved by the Rasch methodology for either of the total scales. This finding indicates that each item of both scales should be scored separately and should be considered as a single item [22]. The main strength of this study was that by using a modern test theory approach, both scales could be improved (e.g., ordered thresholds for the items). However, we could not establish the structural validity and objectivity of either the total scales or the subscales and the total score and subscale scores remained statistically insufficient.

### Limitations and future research

One major limitation of this study was the relatively limited sample size. A sample size of at least 64 to 144 persons is required to achieve 95% confidence that the item calibration is within ± 0.5 logits [40]. Our study sample of 108 patients was within the recommended sample size (i.e., sufficient for the total scales). Subjects with extreme scores were excluded from the analysis because they did not contain information for the estimation of the item and person threshold parameters [36]. Thus, for the subscales, the effective sample size in this study was smaller than the original sample size. The low PSI and the low power of fit had to be taken into account when interpreting the results for the subscales. To derive definitive conclusions about the items and the scales, well-targeted and sample sizes ≥ 250 people are recommended [36, 40]. Therefore, this study must be interpreted as a pilot study. Nonetheless, clinicians and researchers cannot longer rely on both the DSWAL-QoL and aDSWAL-QoL total scores and subscale scores as an indicator of how a patient’s HRQoL is affected by oropharyngeal dysphagia. Until the psychometric properties have been established in a larger sample, we suggest to use the proposed scoring structure (Additional file 1) for each individual item, taking into account to derive only qualitative information from that item. Items suggested to be removed from the total scales and subscales should be interpreted with caution. The psychometric weaknesses of these scales indicate to reconsider the cross-cultural validation process [48] and to evaluate if the translations and adaptations meet accepted standards of cross-cultural validation [4, 22]. We recommend using IRT for further validation of the original SWAL-QoL [11] and all of its translations. Most of the subscales exhibited a lack of sufficient items to allow for the assessment of the unidimensionality of the scale. After all, multiple items enable the improvement of the reliability because random errors of measurement can be averaged out. Multiple items increase the scope of a scale and are less open to variable interpretation [49]. Scales that are too extensive do not function well in routine clinical practice [11] because the patient’s burden in completing multiple items can be onerous and time consuming [50]. With 44 items, both of the SWAL-QoL versions are still long and extensive scales. Therefore, it would be beneficial to create and validate a shorter version. We analyzed the two versions of the SWAL-Qol separately. For future studies, it may be useful to merge the two datasets and perform DIF analysis using the version as a person factor. We used a residual correlation of *r* > 0.30 above the average of all the correlations for the detection of LID [38], although this criterion might be regarded as arbitrary [51]. If a more strict criterion of *r* > 0.20 was used, we might have found more LID.

## Conclusions

This is the first study to examine the structural validity and objectivity of both the DSWAL-QoL and aDSWAL-QoL total scales and subscales and the statistical sufficiency of the total scores and subscale scores using item analysis with the Rasch model. However, the analysis could not establish these psychometric properties because a misfit to the model, multidimensionality, disordered thresholds, DIF and/or LID were found. This analysis with the Rasch model identified areas that require further investigation. Our study highlighted the fact that relying on the DSWAL-QoL and aDSWAL-QoL subscale scores and total scale scores to make conclusions about a person’s dysphagia-related HRQoL should be undertaken with caution before the psychometric requirements have been established. The adjustments suggested by the Rasch model induced scale improvement, as the disordered thresholds were rescaled, the misfit items were removed and the DIF was resolved. Although we were not able to derive definitive conclusions about the items and the scales, this study illustrated the added value of the use of Rasch analysis in the detection of the psychometric strengths and weaknesses of these rating scales. Therefore, this study can be viewed as an essential step forward toward the further improvement of these scales.

## Additional files

Additional file 1: Scoring structure after the adjustments as suggested by the Rasch model. (DOCX 22 kb)

Additional file 2: Representation of the 5-point response categories for the different subscales of the aDSWAL-QoL. These pictures demonstrate how the 5-point response categories for the different subscales are presented in the aDSWAL-QoL with respect to the real size. Note that the translation of the response format into English can slightly deviate from the formulation in the Flemish language. (DOCX 149 kb)

## Abbreviations

AAT: Akense Afasie Test
aDSWAL-QoL: adjusted DSWAL-QoL
ANOVA: Analysis of variance
CTT: Classical test theory
df: Degrees of freedom
DIF: Differential item functioning
DSWAL-QoL: Dutch version of the Swallowing Quality-of-Life
Dys: Patients suffering from dysphagia
DysLC: Patients with dysphagia accompanied by language impairment and/or cognitive disorders
EORTC QLQ- PR25: European Organization for Research and Treatment of Cancer Quality of Life Questionnaire-prostate specific 25-item
ext: Extreme
FEES: Fiberoptic Endoscopic Evaluation of Swallowing
FR: Fit residual
HRQoL: Health-related quality of life
ICC: Intraclass correlation coefficient
IRT: Item response theory
LC: Patients suffering from language impairment and/or cognitive disorders without the presence of dysphagia
LCI: Lower confidence interval
LID: Local item dependence
MASA: Mann Assessment of Swallowing Ability
MMSE: Mini Mental State Examination
PCA: Principal component analysis
PREE: Patient-Rated Elbow Evaluation
PRO: Patient-reported outcome
PSI: Person separation index
SD: Standard deviation
SE: Standard error
SGRQ: St. George’s Respiratory Questionnaire
SLP: Speech-language pathologist
SWAL-QoL: Swallowing Quality-of-Life

## References

[1] Timmerman AA, Speyer R, Heijnen B, Klijn-Zwijnenberg IR (2014). Psychometric characteristics of health-related quality-of-life questionnaires in oropharyngeal dysphagia. Dysphagia.

[2] Sneeuw KC, Sprangers MA, Aaronson NK (2002). The role of health care providers and significant others in evaluating the quality of life of patients with chronic disease. J Clin Epidemiol.

[3] Wheeler A, McKenna B, Madell D, Harrison J, Prebble K, Larsson E (2015). Self-reported health-related quality of life of mental health service users with serious mental illness in New Zealand. J Prim Health Care.

[4] Mokkink LB, Terwee CB, Patrick DL, Alonso J, Stratford PW, Knol DL (2010). The COSMIN checklist for assessing the methodological quality of studies on measurement properties of health status measurement instruments: an international Delphi study. Qual Life Res.

[5] Cano SJ, Hobart JC (2011). The problem with health measurement. Patient Prefer Adherence.

[6] Orlandoni P, Jukic PN (2016). Health-related quality of life and functional health status questionnaires in oropharyngeal dysphagia. J Aging Res Clin Pract.

[7] Vanderwegen J, Van Nuffelen G, De Bodt M (2013). The validation and psychometric properties of the Dutch version of the swallowing quality-of-life questionnaire (SWAL-QoL). Dysphagia.

[8] Finizia C, Rudberg I, Bergqvist H, Rydén A (2012). A cross-sectional validation study of the Swedish version of SWAL-QoL. Dysphagia.

[9] Zraick RI, Atherson SR, Ham BK (2012). Readability of patient-reported outcome questionnaires for use with persons with swallowing disorders. Dysphagia.

[10] de Vet HCW, Terwee CB, Mokkink LB, Knol DL (2011). Measurements in medicine. A practical guide.

[11] McHorney CA, Robbins J, Lomax K, Rosenbek JC, Chignell K, Kramer AE (2002). The SWAL-QOL and SWAL-CARE outcomes tool for oropharyngeal dysphagia in adults: III. Documentation of reliability and validity. Dysphagia.

[12] Speyer R, Heijnen BJ, Baijens LW, Vrijenhoef FH, Otters EF, Roodenburg N (2011). Quality of life in oncological patients with oropharyngeal dysphagia: validity and reliability of the Dutch verison of the MD Anderson dysphagia inventory and the deglutition handicap index. Dysphagia.

[13] Simpelaere IS, Vanderwegen J, Wouters K, De Bodt M, Van Nuffelen G. Feasibility and Psychometric properties of the Adjusted DSWAL-QoL Questionnaire for Dysphagic Patients with Additional Language and/or Cognitive Impairment: Part I. Dysphagia. 2017. doi:10.1007/s00455-016-9770-2 10.1007/s00455-016-9770-2 28101665

[14] Hendriks J, Fyfe S, Styles I, Skinner SR, Merriman G (2012). Scale construction utilising the Rasch unidimensional measurement model: A measurement of adolescent attitudes towards abortion. Australas Med J.

[15] Petrillo J, Cano SJ, McLeod LD, Coon CD (2015). Using classical test theory, item response theory, and Rasch measurement theory to evaluate patient-reported outcome measures: a comparison of worked examples. Value Health.

[16] Lo C, Liang WM, Hang LW, Wu TC, Chang YJ, Chang CH (2015). A psychometric assessment of the St. George’s respiratory questionnaire in patients with COPD using Rasch model analysis. Health Qual Life Outcomes.

[17] Kreiner S (2007). Validity and objectivity: reflections on the role and nature of Rasch models. Nordic Psychol.

[18] Tennant A, Conaghan PG (2007). The Rasch measurement model in rheumatology: What is it and why use it? When should it be applied, and what should one look for in a Rasch paper?. Arthritis Rheum.

[19] Hagell P (2014). Testing rating scale unidimensionality using the principal component analysis (PCA)/t-test protocol with the Rasch model: the primacy of theory over statistics. Open J Stat.

[20] Portney LG, Watkins MP (2000). Foundations of clinical research. Applications to practice.

[21] Hagquist C, Bruce M, Gustavsson JP (2009). Using the Rasch model in nursing research: an introduction and illustrative sample. Int J Nurs Stud.

[22] Christensen KB, Kreiner S, Mesbah M (2013). Rasch models in health.

[23] Chang YJ, Liang WM, Wu HC, Lin HC, Wang JY, Li TC (2012). Psychometric evaluation of the Taiwan Chinese version of the EORTC QLQ-PR25 for HRQoL assessment in prostate cancer patients. Health Qual Life Outcomes.

[24] Vincent JI, MacDermid JC, King GJW, Grewal R (2015). Rasch analysis of the patient-rated elbow evaluation questionnaire. Health Qual Life Outcomes.

[25] Mann G (2002). The Mann assessment of swallowing ability.

[26] Langmore S. Endoscopic evaluation of oral and pharyngeal phases of swallowing. GI Motility online. 2006. doi:10.1038/gimo28.

[27] Graetz P, De Bleser R, Willmes K (1992). Akense Afasie test. Nederlandse versie.

[28] Folstein MF, Folstein SE, McHugh PR (1975). Mini-Mental State: A practical method for grading the cognitive state of patients for the clinician. J psychiat Res.

[29] O’Bryant SE, Humphreys JD, Smith GE, Ivnik RJ, Graff-Radford NR, Petersen RC (2008). Detecting dementia with the mini-mental state examination (MMSE) in highly educated individuals. Arch Neurol.

[30] Kukull WA, Larson EB, Teri L, Bowen J, McCormick W, Pfanschmidt ML (1994). The mini-mental state examination score and the clinical diagnosis of dementia. J Clin Epidemiol.

[31] McHorney CA, Bricker DE, Robbins J, Kramer AE, Rosenbek JC, Chignell KA (2000). The SWAL-QoL outcomes tool for oropharyngeal dysphagia in adults: II. Item reduction and preliminary scaling. Dysphagia.

[32] Hansen T, Lambert H, Faber J (2012). Validation of the Danish version of the McGill ingestive skills assessment using classical test theory and the Rasch model. Dis Rehabil.

[33] Wright BD (1998). Model selection: rating scale model (RSM) or partial credit model (PCM)?. Rasch Meas Trans.

[34] Andrich D, Lyne A, Sheridan B, Luo G (2010). RUMM2030.

[35] Pallant JF, Tennant A (2007). An introduction to the Rasch measurement model: an example using the hospital anxiety and depression scale (HADS). Br J Clin Psychol.

[36] Chen WH, Lenderking W, Jin Y, Wyrwich W, Gelhorn H, Revicki DA (2014). Is Rasch model analysis applicable in small sample size pilot studies for assessing item characteristics? An example using PROMIS pain behavior item bank data. Qual Life Res.

[37] Kutlay Ş, Küçükdeveci AA, Elhan AH, Öztuna D, Koç N, Tennant A (2011). Validation of the World Health Organization disability assessment schedule II (WHODAS-II) in patients with osteoarthritis. Rheumatol Int.

[38] Zucca A, Lambert SD, Boyes AW, Pallant JF (2012). Rasch analysis of the mini-mental adjustment to cancer scale (Mini-MAC) among a heterogeneous sample of long-term cancer survivors: a cross-sectional study. Health Qual Life Outcomes.

[39] Marais I, Andrich D (2008). Formalizing dimension and response violations of local independence in the unidimensional Rasch model. J Appl Meas.

[40] Linacre JM (1994). Sample size and item calibration stability. Rasch Meas Trans.

[41] Chimi CJ, Russell DL. The Likert scale: a proposal for improvement using quasi-continuous variables. In: The proceedings of the information systems education conference. 2009. http://citeseerx.ist.psu.edu/viewdoc/download?doi=10.1.1.467.646&rep=rep1&type=pdf. Accessed Aug 2016.

[42] McKhann GM, Knopman DS, Chertkow H, Hyman BT, Jack CR, Kawas CH (2011). The diagnosis of dementia due to Alzheimer’s disease: recommendations from the National Institute on Aging-Alzheimer’s Association workgroups on diagnostic guidelines for Alzheimer’s disease. Alzheimers Dement.

[43] González-Romá V, Espejo B (2003). Testing the middle response categories «Not sure», «In between» and «?» in polytomous items. Psicothema.

[44] Whitley BE, Kyte ME (2013). Principles of research in behavioural science.

[45] Bogaerdt HC, Speyer R, Baijens LW, Fokkens WJ (2009). Cross-cultural adaptation and validation of the Dutch version of SWAL-QoL. Dysphagia.

[46] Rummlab. Cronbach’s alpha and the Person Separation Index (PSI). http://www.rummlab.com.au/rmrelidx2030. Accessed June 2016.

[47] Holgado-Tello FP, Chacón-Moscoso S, Barbero-García I, Vila-Abad E (2010). Polychoric versus Pearson correlations in exploratory and confirmatory factor analysis of ordinal variables. Qual Quant.

[48] Cordier R, Joosten A, Clavé P, Schindler A, Bülow M, Demier N, et al. Evaluating the Psychometric Properties of the Eating Assessment Tool (EAT-10) Using Rasch Analysis. Dysphagia. 2016. doi:10.1007/s00455-016-9754-2. 10.1007/s00455-016-9754-2 27873090

[49] Hilari K, Boreham LD (2013). Visual analogue scales in stroke: what can they tell us about health-related quality of life?. BMJ Open.

[50] Peeters JB, Daudey L, Heijdra YF, Molema J, Dekhuijzen PN, Vercoulen JH (2009). Development of a battery of instruments for detailed measurement of health status in patients with COPD in routine care: the Nijmegen Clinical Screening Instrument. Qual Life Res.

[51] Fayers PM, Machin D (2015). Quality of life: the assessment, analysis and reporting of patient-reported outcomes.

