# Supplementary material for: Validation of the Dutch version of the Swallowing Quality-of-Life Questionnaire (DSWAL-QoL) and the adjusted DSWAL-QoL (aDSWAL-QoL) using item analysis with the Rasch model: a pilot study
Source: Health Qual Life Outcomes. 2017 Apr 7;15:66. doi: 10.1186/s12955-017-0639-3 (PMC5383953; doi:10.1186/s12955-017-0639-3)
Supplement: Supplementary file 1 — Scoring structure after the adjustments as suggested by the Rasch model. (DOCX 22 kb) [file 12955_2017_639_MOESM1_ESM.docx]

| **Additional file 1.** Scoring structure after the adjustments as suggested by the Rasch model | | | | | | |
| --- | --- | --- | --- | --- | --- | --- |
|  |  |  | DSWAL-QoL | | aDSWAL-QoL | |
|  | Item content | Subscale | Total scale | Subscales | Total scale | Subscales |
| 1 | Difficult dealing | Burden | 0,1,1,1,2 | 0,0,1,1,2 | 0,1,1,1,2^a^ | **0,1,2,3,4** |
| 2 | Major distraction | Burden | 0,1,1,1,2 | 0,0,1,1,2 | 0,1,1,1,2^a^ | **0,1,2,3,4** |
| 3 | Longer time to eat | Eating duration | 0,1,1,1,2 | 0,1,1,1,2 | 0,1,1,1,2 | 0,1,2,2,3^a^ |
| 4 | Takes forever to eat | Eating duration | 0,1,1,1,2 | 0,0,1,1,2 | 0,1,1,1,2 | **0,1,2,3,4** |
| 5 | Don't care if I eat or not | Eating desire | ~~0,0,0,1,1~~ | 0,1,1,1,2 | ~~0,1,1,1,2~~ | 0,1,1,1,2 |
| 6 | Don't enjoy | Eating desire | ~~0,0,0,1,1~~ | 0,1,1,1,2 | ~~0,1,1,1,2~~ | 0,1,1,2,3 |
| 7 | Rarely hungry | Eating desire | ~~0,0,0,1,1~~ | 0,1,1,1,2 | ~~0,1,1,1,2~~ | 0,1,1,2,3 |
| 8 | Coughing | Symptoms | **0,1,2,3,4** | **0,1,2,3,4** | **0,1,2,3,4** | **0,1,2,3,4** |
| 9 | Choking on food | Symptoms | 0,1,1,1,2 | 0,1,1,1,2 | 0,0,1,1,2 | 0,1,1,2,3 |
| 10 | Choking on liquids | Symptoms | **0,1,2,3,4** | **0,1,2,3,4** | 0,0,1,1,2 | **0,1,2,3,4** |
| 11 | Thick saliva, phlegm | Symptoms | 0,1,1,1,2 | 0,1,2,2,3 | 0,1,1,1,2 | 0,0,1,2,2 |
| 12 | Gagging | Symptoms | 0,1,1,1,2 | 0,1,2,2,3 | 0,0,0,1,2 | **0,1,2,3,4** |
| 13 | Excess saliva, phlegm | Symptoms | 0,1,1,1,2 | 0,1,1,1,2 | 0,1,1,2,2 | 0,1,2,2,3 |
| 14 | Clear throat | Symptoms | 0,1,1,1,2 | 0,1,1,1,2 | 0,1,1,1,2 | 0,1,1,1,2 |
| 15 | Drooling | Symptoms | 0,1,1,1,2 | 0,1,1,1,2 | 0,1,1,1,2 | **0,1,2,3,4** |
| 16 | Problem chewing | Symptoms | 0,1,1,1,2 | 0,1,2,2,3 | 0,1,2,2,3 | **0,1,2,3,4** |
| 17 | Food stick throat | Symptoms | 0,1,1,1,2 | 0,1,2,2,3 | 0,1,1,1,2 | 0,1,2,2,3 |
| 18 | Food stick mouth | Symptoms | **0,1,2,3,4** | 0,1,1,1,2 | 0,1,1,1,2 | 0,1,1,2,3 |
| 19 | Food/liquid dribble from mouth | Symptoms | 0,1,1,1,2 | 0,1,1,1,2 | 0,1,1,1,2 | 0,1,1,1,2 |
| 20 | Food/liquid dribble from nose | Symptoms | 0,1,1,1,2 | 0,1,2,2,3, | 0,0,1,1,2 | 0,1,2,2,3 |
| 21 | Cough out of mouth when food stuck | Symptoms | 0,1,1,1,2 | **0,1,2,3,4** | 0,1,1,1,2 | 0,0,1,1,2 |
| 22 | Figure can - can't eat | Food selection | 0,1,1,1,2 | 0,1,2,2,3 | 0,1,1,1,2 | 0,1,1,1,2 |
| 23 | Difficult dealing | Food selection | 0,1,1,1,2 | 0,1,2,2,3 | 0,1,1,1,2 | 0,1,1,1,2 |
| 24 | Hard understand me | Communication | 0,1,1,1,2 | **0,1,2,3,4** | 0,1,1,1,2 | 0,0,1,2,2 |
| 25 | Hard speaking clear | Communication | 0,1,1,1,2 | **0,1,2,3,4** | 0,1,1,1,2 | **0,1,2,3,4** |
| 26 | Afraid choking foods | Fear | 0,1,1,1,2 | 0,1,1,1,2^a^ | 0,1,1,1,2 | 0,1,1,2,2 |
| 27 | Afraid pneumonia | Fear | 0,1,1,1,2 | 0,1,1,1,2 | 0,1,1,1,2^a^ | **0,1,2,3,4** |
| 28 | Afraid choking liquids | Fear | 0,1,1,1,2 | 0,1,1,1,2 | 0,1,1,1,2 | 0,0,1,2,2 |
| 29 | Never know when choke | Fear | 0,1,1,1,2 | 0,1,1,1,2 | ~~0,1,1,1,2~~ | ~~0,0,1,2,2~~ |
| 30 | Depressed | Mental health | 0,1,1,1,2 | 0,0,1,1,2 | 0,1,1,1,2 | 0,1,1,2,2 |
| 31 | Impatient dealing | Mental health | 0,1,1,1,2 | **0,1,2,3,4** | 0,1,1,1,2 | 0,0,1,1,2 |
| 32 | So careful annoy | Mental health | 0,1,1,1,2 | **0,1,2,3,4** | ~~0,1,1,1,2~~ | **0,1,2,3,4** |
| 33 | Frustrated | Mental health | 0,1,1,1,2 | **0,1,2,3,4** | 0,1,1,1,2 | 0,1,1,2,3 |
| 34 | Discouraged | Mental health | ~~0,1,1,1,2~~ | **0,1,2,3,4** | 0,1,1,1,2 | 0,0,1,1,2 |
| 35 | Do not go out | Social functioning | 0,1,1,1,2 | 0,1,1,1,2 | 0,1,1,1,2 | 0,1,1,1,2 |
| 36 | Hard social life | Social functioning | 0,1,1,1,2 | 0,1,1,1,2 | 0,1,1,1,2 | 0,1,1,1,2 |
| 37 | Change work activity | Social functioning | 0,1,1,1,2 | 0,1,1,1,2 | 0,1,1,1,2 | 0,1,1,1,2 |
| 38 | Dislike social gathering | Social functioning | 0,1,1,1,2 | 0,1,1,1,2 | 0,1,1,1,2 | 0,1,1,1,2 |
| 39 | Role change | Social functioning | 0,1,1,1,2 | 0,1,1,1,2 | 0,1,1,1,2 | 0,1,1,1,2 |
| 40 | Feel exhausted | Fatigue | ~~0,1,1,1,2~~ | 0,0,1,2,2 | ~~0,1,1,1,2~~ | **0,1,2,3,4** |
| 41 | Feel weak | Fatigue | ~~0,1,1,1,2~~ | 0,0,1,2,2 | ~~0,1,1,1,2~~ | **0,1,2,3,4** |
| 42 | Feel tired | Fatigue | 0,1,1,1,2 | 0,0,1,2,2 | 0,1,1,1,2 | **0,1,2,3,4** |
| 43 | Trouble falling asleep | Sleep | 0,1,1,1,2 | 0,1,1,1,2 | 0,1,1,1,2^a^ | 0,0,1,1,2 |
| 44 | Trouble staying asleep | Sleep | 0,1,1,1,2 | 0,1,1,1,2 | 0,1,1,1,2^a^ | **0,1,2,3,4** |
| Note: The score range from 1 to 5 is transformed into the range of 0 to 4 in the RUMM2030. Bold indicates that the original scoring structure worked as intended. Items crossed out indicate that these items were removed from the scale.  ^a^ items were split for DIF. | | | | | | |
