# Supplementary material for: Validation of the Dutch version of the Swallowing Quality-of-Life Questionnaire (DSWAL-QoL) and the adjusted DSWAL-QoL (aDSWAL-QoL) using item analysis with the Rasch model: a pilot study
Source: Health Qual Life Outcomes. 2017 Apr 7;15:66. doi: 10.1186/s12955-017-0639-3 (PMC5383953; doi:10.1186/s12955-017-0639-3)
Supplement: Supplementary file 2 — Representation of the 5-point response categories for the different subscales of the aDSWAL-QoL. These pictures demonstrate how the 5-point response categories for the different subscales are presented in the aDSWAL-QoL with respect to the real size. Note that the translation of the response format into English can slightly deviate from the formulation in the Flemish language. (DOCX 149 kb) [file 12955_2017_639_MOESM2_ESM.docx]

**Additional file 2.** Representation of the 5-point response categories for the different subscales of the aDSWAL-QoL

The General burden, Eating duration, Eating desire, and Food selection subscales used the following response format:

| √√  yes | √  quite a bit | √X  somewhat | X  a little | X X  no |
| --- | --- | --- | --- | --- |

| **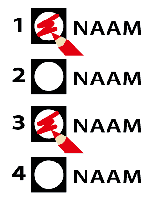** | **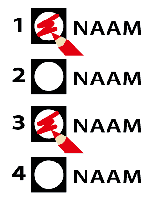** | **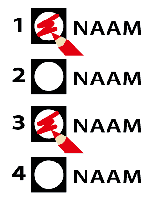** | **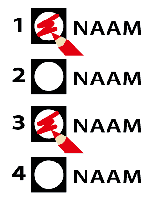** | **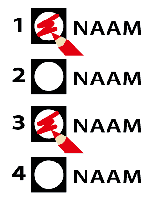** |
| --- | --- | --- | --- | --- |

The Social functioning subscale used the following response format:

| √√  yes | √  quite a bit | ?  don’t know | X  a little | X X  no |
| --- | --- | --- | --- | --- |

| **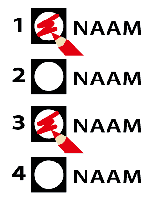** | **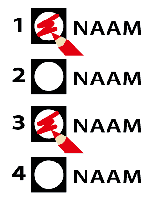** | 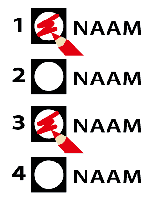 | **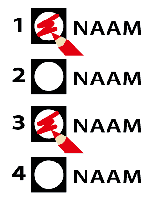** | **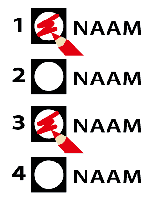** |
| --- | --- | --- | --- | --- |

The Symptoms, Communication, Fear of eating, Mental health, Fatigue and Sleep subscales used the following response format:

| 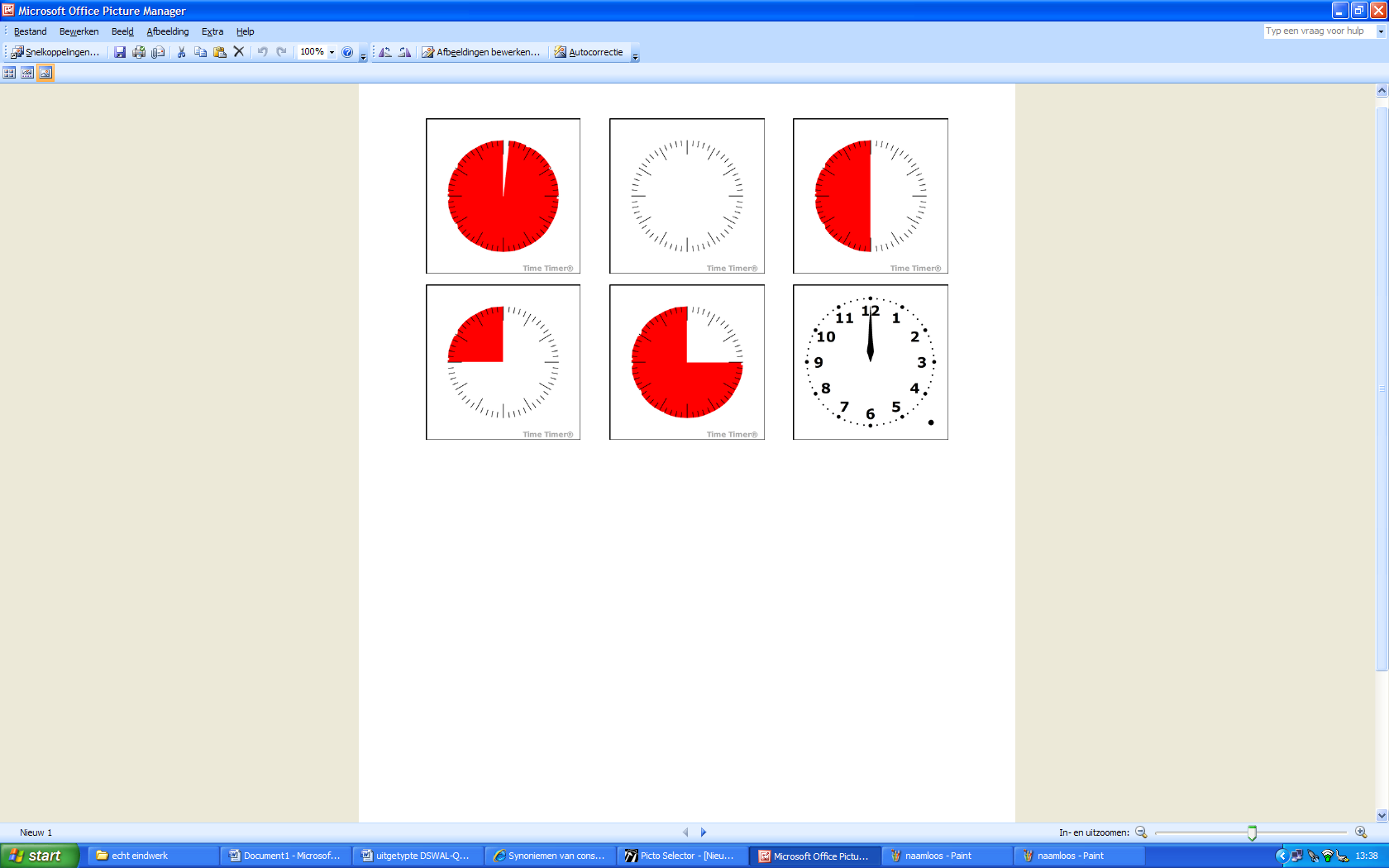  almost always | 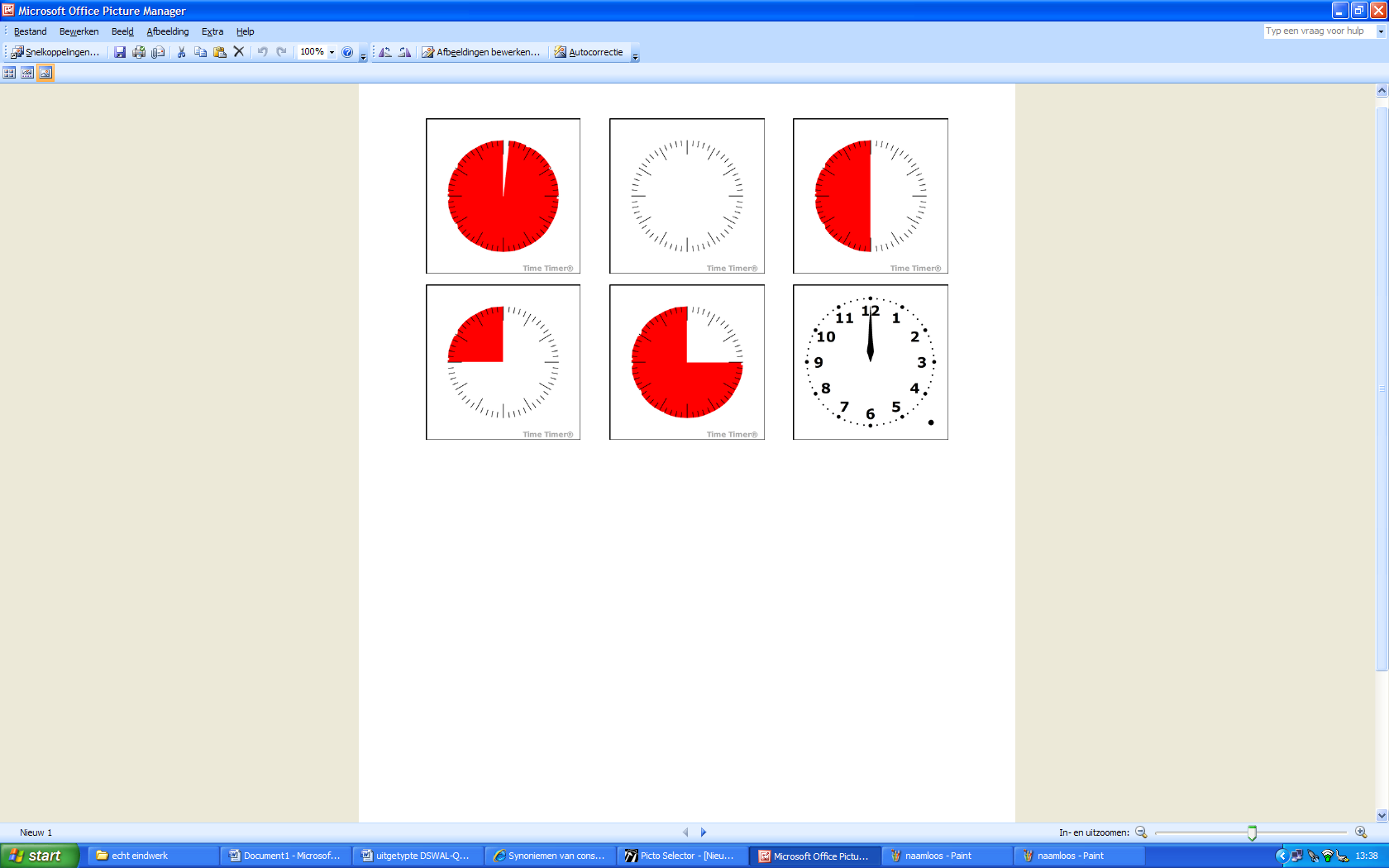  often | 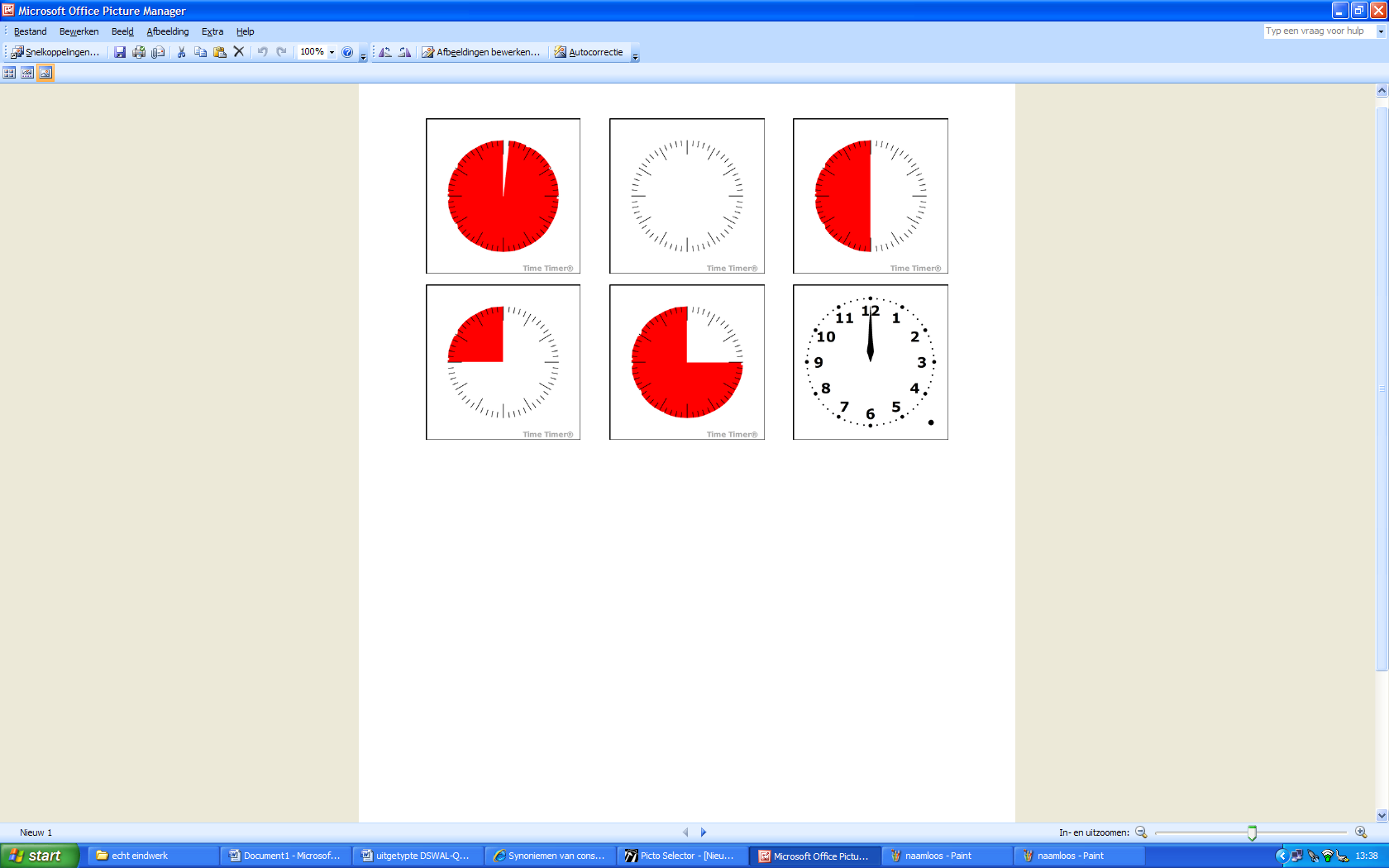  sometimes | 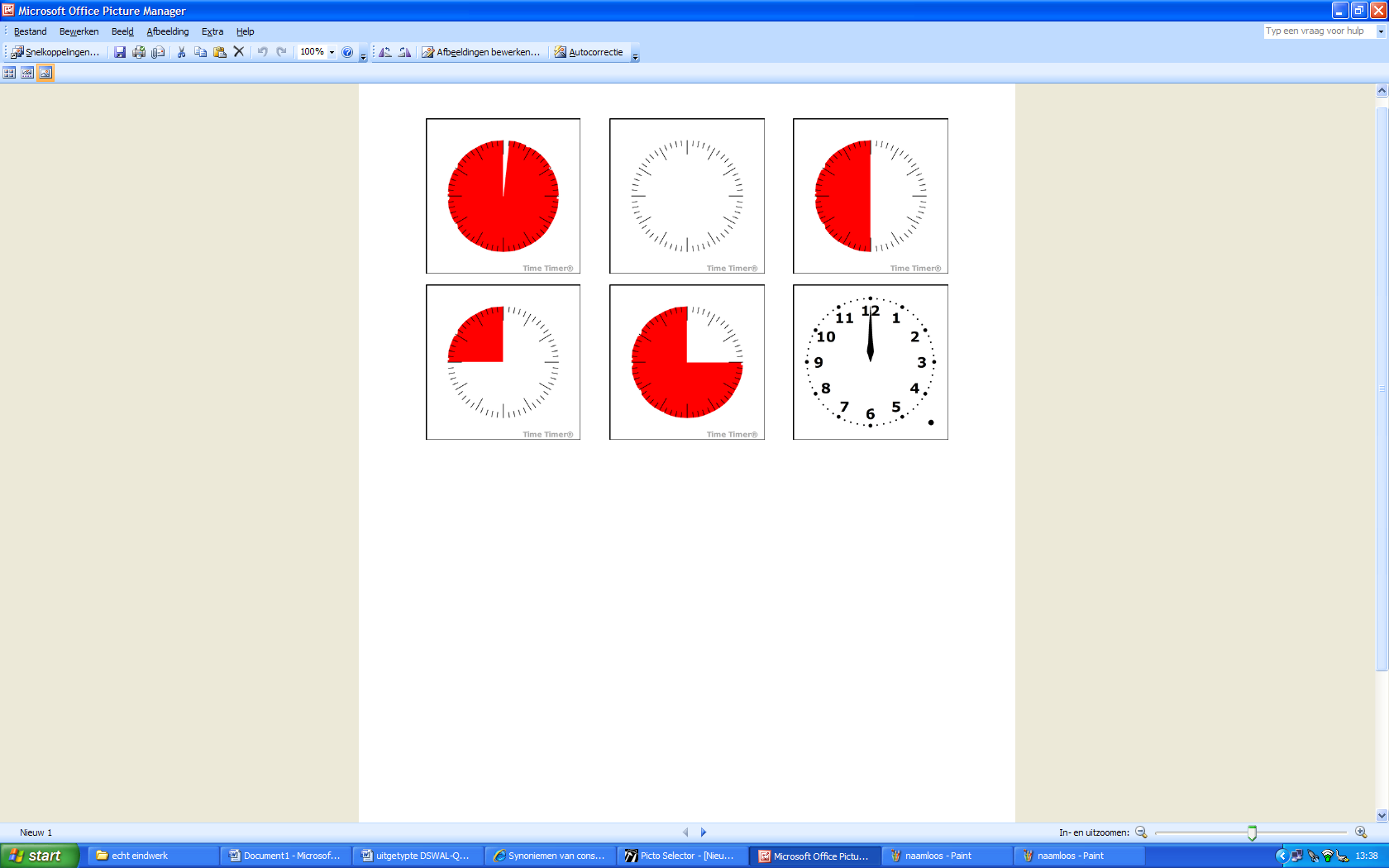  hardly ever | 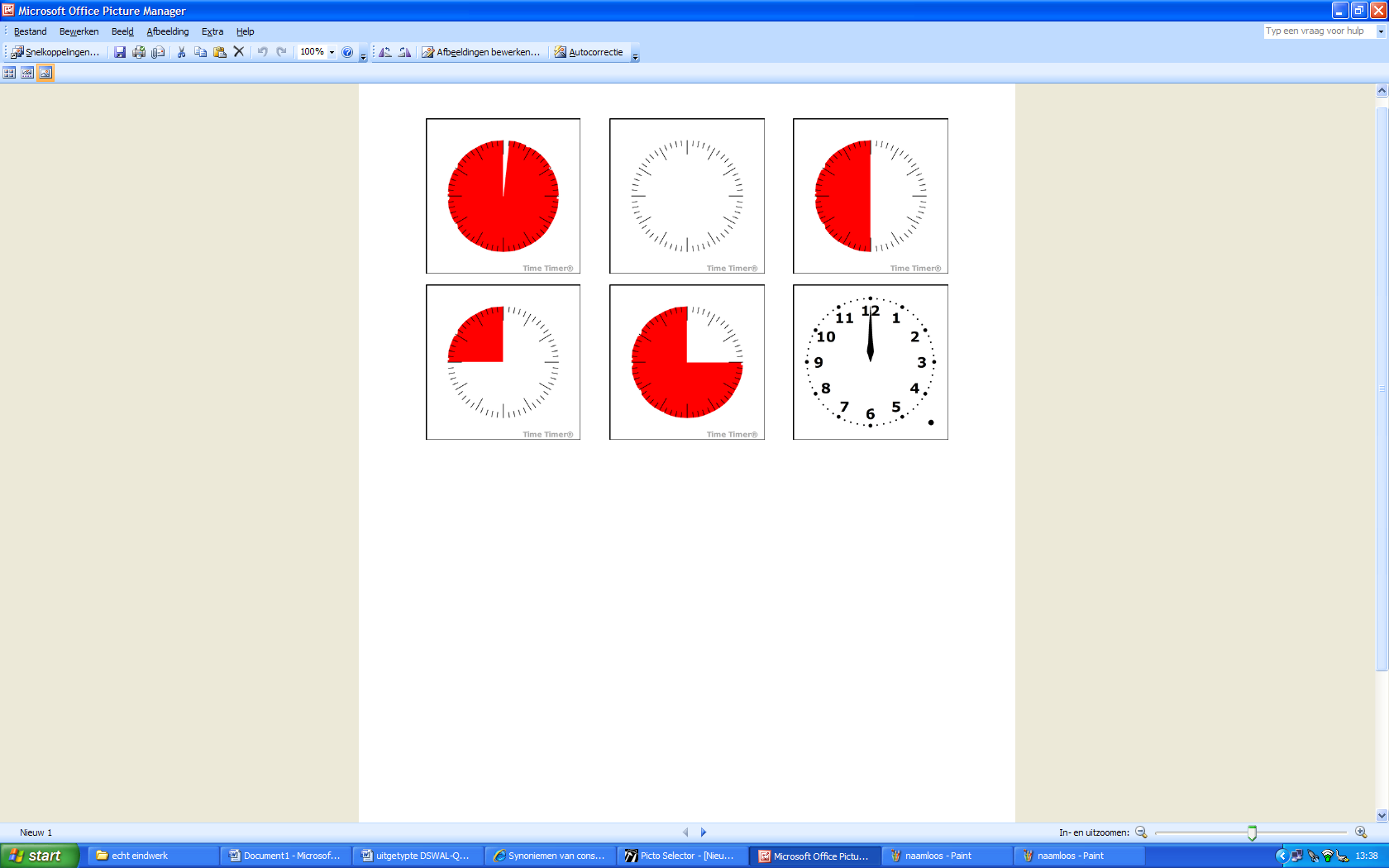  never |
| --- | --- | --- | --- | --- |
| **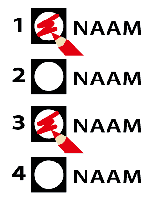** | **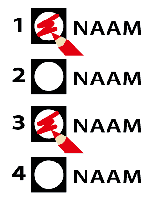** | **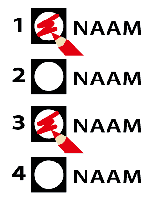** | **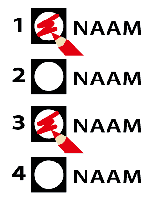** | **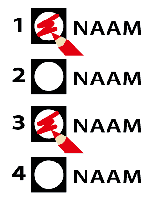** |
